# Timing of Newborn Blood Collection Alters Metabolic Disease Screening Performance

**DOI:** 10.3389/fped.2020.623184

**Published:** 2021-01-20

**Authors:** Gang Peng, Yishuo Tang, Tina M. Cowan, Hongyu Zhao, Curt Scharfe

**Affiliations:** ^1^Department of Genetics, Yale University School of Medicine, New Haven, CT, United States; ^2^Department of Biostatistics, Yale University School of Public Health, New Haven, CT, United States; ^3^Department of Pathology, Stanford University School of Medicine, Stanford, CA, United States

**Keywords:** newborn screening, inborn errors of metabolism, age at blood collection, gestational age, sex, race and ethnicity

## Abstract

Blood collection for newborn genetic disease screening is preferably performed within 24–48 h after birth. We used population-level newborn screening (NBS) data to study early postnatal metabolic changes and whether timing of blood collection could impact screening performance. Newborns were grouped based on their reported age at blood collection (AaBC) into early (12–23 h), standard (24–48 h), and late (49–168 h) collection groups. Metabolic marker levels were compared between the groups using effect size analysis, which controlled for group size differences and influence from the clinical variables of birth weight and gestational age. Metabolite level differences identified between groups were correlated to NBS data from false-positive cases for inborn metabolic disorders including carnitine transport defect (CTD), isovaleric acidemia (IVA), methylmalonic acidemia (MMA), and phenylketonuria (PKU). Our results showed that 56% of the metabolites had AaBC-related differences, which included metabolites with either decreasing or increasing levels after birth. Compared to the standard group, the early-collection group had elevated marker levels for PKU (phenylalanine, Cohen's *d* = 0.55), IVA (C5, Cohen's *d* = 0.24), MMA (C3, Cohen's *d* = 0.23), and CTD (C0, Cohen's *d* = 0.23). These findings correlated with higher false-positive rates for PKU (*P* < 0.05), IVA (*P* < 0.05), and MMA (*P* < 0.001), and lower false-positive rate for CTD (*P* < 0.001) in the early-collection group. Blood collection before 24 h could affect screening performance for some metabolic disorders. We have developed web-based tools integrating AaBC and other variables for interpretive analysis of screening data.

## Introduction

The timing of blood sampling and postnatal age are important parameters for accurately interpreting test results for newborn screening. The Clinical and Laboratory Standards Institute (CLSI) recommends blood spot collection on filter paper for genetic disease screening between 24 and 48 h of age (1). NBS programs have implemented different cutoff values for some metabolic disorders detectable by tandem mass spectrometry (MS/MS) depending on the infant's age (in hours) at blood collection (AaBC). Blood spots drawn too early may impair the detection of some metabolic disorders due to the infant's biochemical transition from a mother-dependent to an autonomous state, while collection after 48 h of age could delay diagnosis and initiation of treatment for some infants (2, 3). Under some circumstances such as birth stress, prematurity, low birth weight or infant disease, blood sampling could be delayed. Several studies examining the association between AaBC and MS/MS-based screening have focused on a single or a few metabolic analytes or groups of metabolic disorders (4–9). In addition to AaBC, metabolic changes have also been associated with other confounding clinical variables such as gestational age (GA), birth weight (BW), sex, season of birth and race/ethnicity status reported by the parents (10–17).

In this study, we used population-level data reported by the California NBS program to study early postnatal metabolic changes and whether AaBC could impact screening performance for inborn metabolic disorders on the Recommended Universal Screening Panel (RUSP) (18). Since both GA and BW are known to influence metabolic marker levels (13, 14, 16), we controlled for the influence of these covariates in the analysis of metabolite levels between AaBC groups. We also studied the influence of race/ethnicity status and total parenteral nutrition (TPN) on metabolic analyte levels across different AaBC timepoints ranging from 12 to 168 h after birth. Finally, the influence of the AaBC on false-positive newborn screens was investigated. The identified AaBC-related differences in metabolite levels were correlated to false-positive cases for eleven inborn metabolic disorders. Based on these findings, web-based tools were developed to aid the interpretation of NBS data in relation to AaBC (http://rusptools.shinyapps.io/AaBC/), and to support development of algorithms that incorporate information on a variety of clinical variables in genetic disease screening.

## Materials and Methods

### Data Summary

NBS data from 503,935 screen-negative singleton babies born between 2013-2017 were analyzed. The cohort was selected at random by the California NBS program. The data included 41 metabolic analytes measured by MS/MS (19) and six clinical variables of birth weight (BW), gestational age (GA), sex, race/ethnicity, total parenteral nutrition (TPN), and age at blood collection (AaBC). Infants with unknown AaBC or blood collection before 12 or after 168 h were removed from the analysis as were infants with BW smaller than 1,000 g or larger than 5,000 g, or with GA smaller than 28 weeks or larger than 42 weeks, which resulted in 500,539 newborns remaining for downstream analysis (Supplementary Table 1). In addition, we analyzed data from screen-positive newborns for eleven inborn metabolic disorders reported by the California NBS program. This cohort consisted of confirmed true-positive cases and of first-tier false-positive cases for argininosuccinic aciduria (ASA), citrullinemia type 1 (CIT-I), citrullinemia type 2 (CIT-II), carnitine transporter deficiency (CTD), homocystinuria (HCY), isovaleric acidemia (IVA), methylmalonic acidemia (MMA), propionic acidemia (PA), phenylketonuria (PKU), ornithine transcarbamylase deficiency (OTCD), and very long-chain acyl-CoA dehydrogenase deficiency (VLCADD) (Table 1). All screen-positive newborns were collected between 2013 to 2017 except for MMA, OTCD, and VLCADD collected between 2005 to 2015. This study was overseen by the institutional review boards at Yale University (protocol #1505015917), Stanford University (protocol #30618), and the State of California Committee for the Protection of Human Subjects (protocol #13-05-1236).

**Table 1:** Correlation of metabolite levels between screen-negatives and false-positives at early, standard and late AaBC.

		**Effect Size^∧^**		**Number of False Positives (%)**		
**Disorder**	**Marker (20)**	**Early**	**Late**	**Early**	**Standard**	**Late**
CTD	C0↓	**0.23^+^**	−0.015	44 (9.3%) E: 102^***^	381 (80.9%) E: 347	46 (9.8%) E: 22^***^
PA	C3↑	**0.23^#^**	−0.11	29 (29.6%) E: 21	65 (66.3%) E: 72	4 (4.1%) E: 5
MMA	C3↑	**0.23^+^**	−0.11	66 (34.7%) E: 41^***^	97 (51.1%) E: 140	27 (14.2%) E: 9^***^
IVA	C5↑	**0.24^+^**	**0.31^&^**	12 (41.4%) E: 6^*^	16 (55.2%) E: 21	1 (3.5%) E: 1
VLCADD	C14:1↑	−0.04	–**0.26^&^**	20 (15.2%) E: 29	97 (73.5%) E: 97	15 (11.4%) E: 6^**^
CITR^$^	Citrulline↑	0.06	–**0.28^&^**	19 (19.6%) E: 21	68 (70.1%) E: 71	10 (10.3%) E: 4^*^
OTCD	Citrulline↓	0.06	–**0.28^+^**	27 (11.0%) E: 53	129 (52.7%) E: 181	89 (36.3%) E: 11^***^
HCY	Methionine↑	**0.50^#^**	–**0.34^#^**	3 (25.0%) E: 3	9 (75.0%) E: 9	0 (0.0%) E: 1
PKU	Phenylalanine↑	**0.55^+^**	–**0.41^+^**	51 (31.3%) E: 35^*^	112 (68.7%) E: 120	0 (0.0%) E: 7^*^

$ *CITR includes three disorders (ASA, CIT-I, and CIT-II) detected through elevated citrulline levels*.

* *P < 0.05*;

** *P < 0.01*;

*** *P < 0.001*.

*The percentage difference between false positive and screen negative infants in early and late collection groups was compared using Chi-squared test. There are 90,060 (21.7%), 305,674 (73.7%), and 19,135 (4.6%) screen negative newborns in the early, standard and late collection groups, respectively. E indicates expected number of false positives*.

# *Sample size of false positive cases is relatively small*.

+ Consistent and

& *Inconsistent between Cohen's d and number of false-positives. See for definition in Supplementary Table 3*.

∧ *Effect size analysis using Cohen's d for marker level differences between early (12–23 h) and late (49–168 h) collection-groups compared to the standard group (24–48 h). Cohen's d with absolute value larger than 0.2 in **bold***.

### Analysis of AaBC

**Figure 1:**
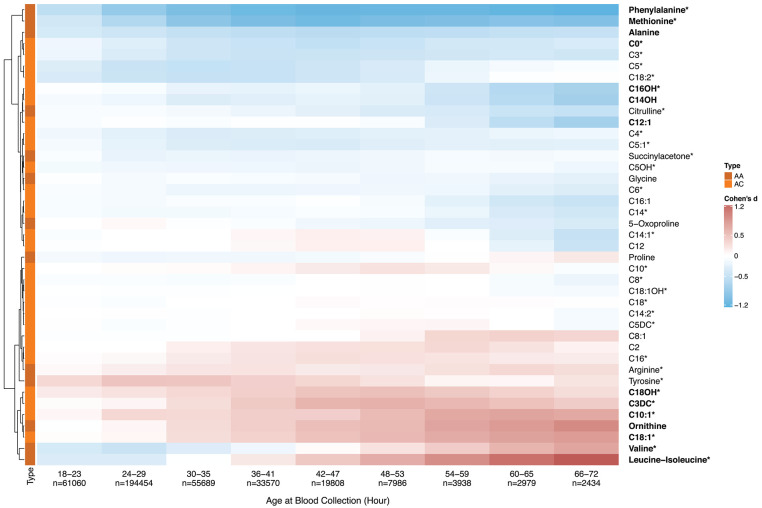
Metabolic analyte levels and age at blood collection. To explore early postnatal metabolic changes, we selected ten infant groups based on AaBC differences with the first group (12–17 h, *n* = 29,000) being defined as a standard for each metabolite. Effect size differences (Cohen's *d*) for all 41 metabolites between each of nine AaBC groups (18–72 h) and the standard (12–17 h) was calculated. Positive Cohen's *d* (in red) indicates elevated metabolite levels, negative Cohen's *d* (in blue) indicates decreased levels compared to the standard. Hierarchically clustering was used to group metabolites into two cluster of either decreasing (on top) or increasing (at bottom) levels after birth. Thirty four percent of the markers showed significant differences between at least one of the nine AaBC groups and the standard group (Cohen's *d* ≥ 0.5), including 30 primary NBS markers of metabolic disorders on the RUSP (18) (labeled with*). AA, amino acid; AC, acylcarnitine.

To reduce the influence from the covariates of GA and BW on metabolite levels (13, 14, 16), our AaBC analysis included 414,869 screen-negative term infants [37–41 weeks] with BW range of 2,500 g to 4,000 g. Infants with positive or unknown TPN status were removed from analysis (21). We first investigated metabolite changes across different AaBC timepoints between 12 to 72 h after birth (*n* = 410,918). Infants were divided into nine AaBC groups of 6 h collection windows except for the last group (66–72 h). AaBC data after 72 h were excluded from analysis due to small sample size. Metabolite levels of 41 MS/MS metabolites in the 12–17 h AaBC group were used as the standard. This choice was made to explore the gradual changes in metabolite levels shortly after birth. We performed effect size analysis using Cohen's d (22) to calculate marker level differences for each of the nine remaining AaBC groups in comparison to the 12–17 h standard group. Cohen's *d* values calculated for each AaBC group were recorded in a data matrix and hierarchical clustering was used to compare AaBC-related profiles between the metabolites (Figure 1).

### Analysis of AaBC in Relation to Other Variables

**Figure 2:**
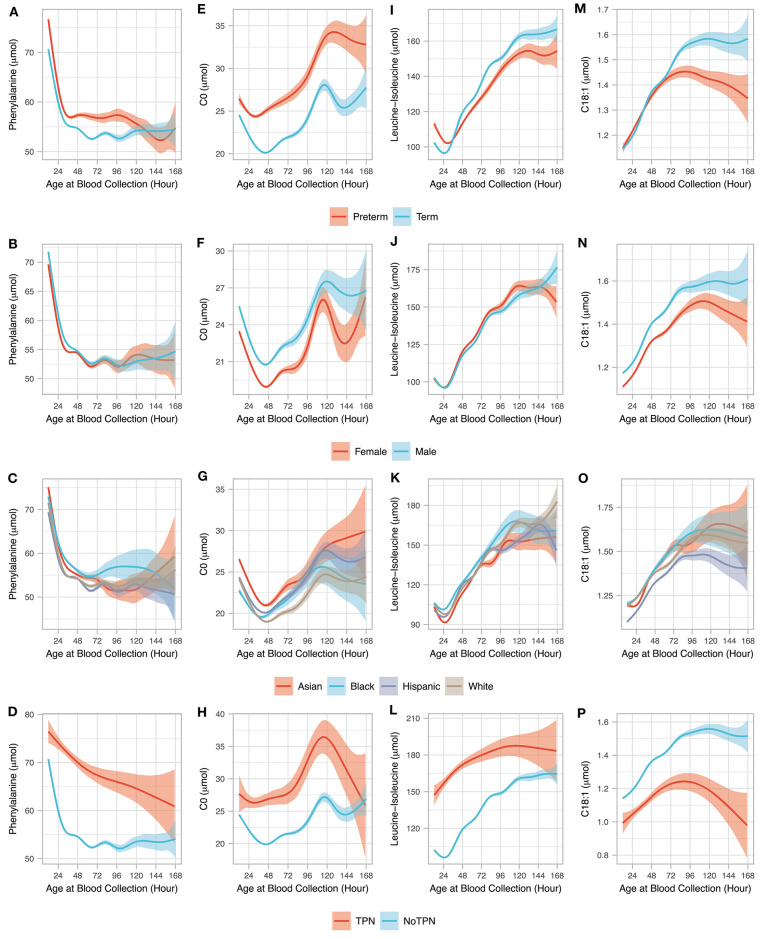
Age at blood collection and clinical variables. The association between AaBC (12–168 h) and four clinical variables (gestational age, sex, race/ethnicity, and total parenteral nutrition) are shown for four metabolites including phenylalanine **(A–D)**, C0 **(E–H)**, leucine-isoleucine **(I–L)**, and C18:1 **(M–P)**. For each of the 4 metabolites, relationship between the different AaBC timepoints are shown for preterm (*n* = 24,447) and term (*n* = 462,806) infants without TPN **(A,E,I,M)**; for male (*n* = 209,855) and female (*n* = 203,799) infants born at term and normal BW and without TPN **(B,F,J,N)**; for infants from different race/ethnicity groupings born at term and normal BW and without TPN including Asian (*n* = 63,528), Black (*n* = 27,301), Hispanic (*n* = 207,499), and White (107,561) **(C,G,K,O)**; and for term infants with normal BW and with (*n* = 1,686) and without TPN (*n* = 414,869). The solid smoothed lines are the mean estimated from a generalized additive model with the shading showing the 95% confidence interval of the mean estimation.

Two metabolites with decreasing (phenylalanine and free carnitine, C0) and two metabolites with increasing (leucine-isoleucine and C18:1) levels between 18 to 72 h after birth were selected to study the influence of clinical variables on metabolite levels (Figure 2). These four primary NBS markers were among the metabolites found with the largest changes related to AaBC. Firstly, for GA, the changes in metabolite levels related to AaBC were compared between preterm (28–36 weeks) and term (37–41 weeks) newborns using a generalized additive model (23). Because BW and GA are highly correlated and GA is a stronger predictive covariate compared to BW (24), we did not control for birth weight in this analysis. Infants with positive or unknown TPN status were removed from analysis. We did not study post-term (>41 weeks) infants due to small samples size (*n* = 159) amongst infants with AaBC between 49 to 168 h (Supplementary Table 2). Secondly, for sex, the changes in metabolite levels related to AaBC were compared between female and male newborns using a generalized additive model (23). In this analysis, we only included infants born at term (37–41 weeks) and with BW between 2,500 g and 4,000 g, while infants with positive or unknown TPN status were removed. Thirdly, for race/ethnicity, the changes in metabolite levels related to AaBC were compared between four major race/ethnicity groups (Asian, Black, Hispanic, and White) using the same data and methods as described above. The race/ethnicity status of the newborn was self-reported by the parents. Of the 503,935 infants studied, 80.1% (*n* = 403,425) were reported as being of Asian, Black, Hispanic, or White origin. Newborns recorded with more than one race/ethnicity (17.8%, *n* = 89,765) were classified according to NBS program guidelines (25) as follows: (a) Hispanic, if reported Hispanic and any other race/ethnicity; (b) Black, if reported Black and any other race/ethnicity except Hispanic; (c) Asian, if reported Asian and any other race/ethnicity except Hispanic and Black; (d) White, if reported White only. All other ethnicities and unknown race/ethnicity were recorded as Other/Unknown (2.1%, *n* = 10,745). Lastly, for TPN, the changes in metabolite levels related to AaBC were compared between newborns without and with TPN.

### Analysis of AaBC-Related Differences and False-Positive Results

**Figure 3:**
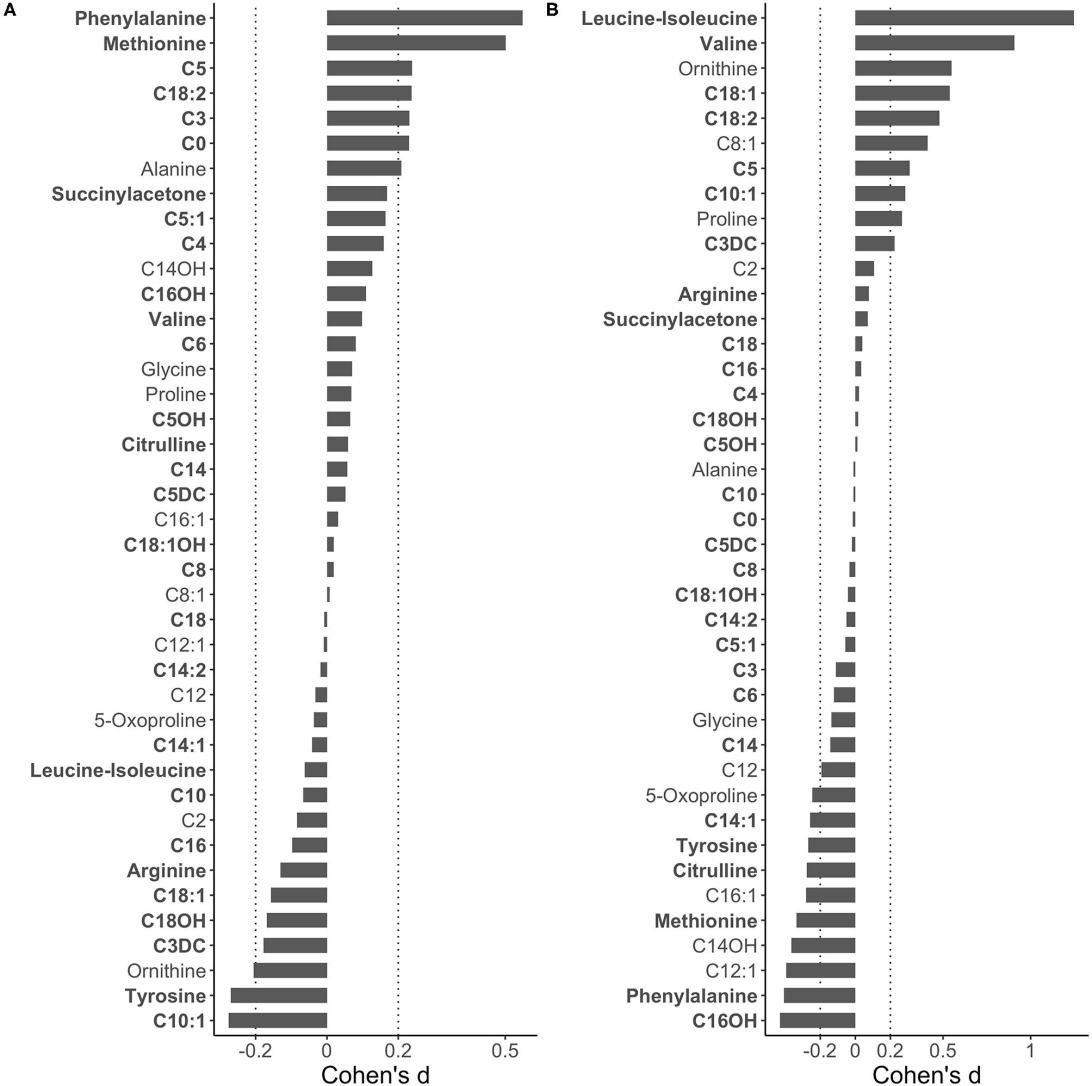
Metabolic difference between early and late blood collection. To identify metabolite level difference related to AaBC, 414,869 screen-negative term infants (37–41 weeks) with BW range of 2,500 g to 4,000 g and without TPN were grouped into early (12–23 h), late (49–168 h) and standard (24–48 h) collection groups. Effect size differences (Cohen's *d*) for each of 41 MS/MS metabolites were calculated between the early and the standard group **(A)** and between the late and standard group **(B)**. In comparison to the standard, positive Cohen's *d* values indicate relatively higher metabolite levels in the early and the late collection groups, respectively. Metabolites are ranked from top to bottom based on Cohen's *d* values with primary NBS disease markers in bold.

The eleven metabolic diseases studied were detected in NBS by elevated (ASA, CIT-I, CIT-II, HCY, IVA, MMA, PA, PKU, and VLCADD) or by decreased (CTD and OTCD) marker levels (Table 1). Here we studied whether timing of blood collection could impact NBS performance for detecting these diseases. Screen-negative newborns were grouped based on their reported AaBC into early (12–23 h), standard (24–48 h), and late (49–168 h) collection groups. We only used data from infants born at term (37 to 41 weeks), birth weight between 2,500 g and 4,000 g, and without TPN to control for the confounding effects of GA, BW, and TPN on marker levels. Data from 414,869 newborns (82.9% of the total) was used in the analysis including 90,060 (21.7%) in the early, 305,674 (73.7%) in the standard, and 19,135 (4.6%) in the late collection groups, respectively. Effect-size analysis using Cohen's *d* (22) for all 41 metabolites was used to compare the early and the late collection-group to the standard group. Metabolites identified in the early or late collection-groups with absolute Cohen's *d* larger than 0.2 were matched to metabolic markers for 11 diseases studied (Figure 3). Only false-positive cases with the same ranges for GA and BW and without TPN as the screen-negatives were selected for analysis. For each disease, the proportion of false positive cases was compared to the proportion of screen negative infants for each of the three AaBC categories using Chi-squared test.

### Statistical Analysis and Online Tool

Statistical analyses, graphs, design of the research and the online tool were performed in R software 3.6.122 with the following R packages: effsize (26), ggplot2 (27), ggsci (28), ggpubr (29), ComplexHeatmap (30), and shiny (31). Effect size analysis using Cohen's *d* (22), which is defined as the difference between two group means divided by the pooled standard deviation, was performed to compare metabolite levels between AaBC groups. Cohen's *d*, which is not influenced by sample size, allowed for direct comparison of metabolite levels between groups with different sample sizes. A novel web-based tool was developed (http://rusptools.shinyapps.io/AaBC/) using the R shiny package (31) for analysis and interpretation of all 41 metabolic analytes and their ratios from 500,539 newborns in relation to AaBC and other covariates (Supplementary Figure 1). A detailed description of the online tool and user guide is provided under Supplementary Material. The source code for the new software is available at GitHub (https://github.com/peng-gang/AaCShiny).

## Results

### Identification of Metabolic Differences Related to AaBC

Population-level MS/MS and timing of blood collection data reported by the California NBS program were used to identify metabolic changes during the first days of life. The 41 MS/MS metabolites were found to cluster into two major groups according to their changing profiles in relation to AaBC (Figure 1). The two clusters showed either decreasing or increasing metabolite levels, while additional changes were observed within each cluster. For example, phenylalanine levels decreased sharply in the first 36 h after birth with very small changes after 48 h, while C16OH decreased steadily from 18 to 72 h after birth. In contrast, leucine-isoleucine levels decreased during the first 30 h and then increased.

We selected four NBS primary markers (two amino acids and two acylcarnitines) that were among the top metabolites identified in Figure 1 to showcase the dynamic metabolic changes associated with AaBC and other clinical variables including GA, sex, race/ethnicity, and TPN (Figure 2). For phenylalanine, preterm infants had higher levels than term infants in the first 108 h after birth. Sex and race/ethnicity status did not have a major effect on phenylalanine levels, while infants under TPN had much higher levels than those without TPN (Figure 2A–D). For free carnitine (C0), its level decreased during the first 48 h except for newborns with TPN, and then increased between 48 and 120 h. GA, sex, race/ethnicity and TPN status had relatively large influence on C0 levels with preterm, male, Asian and newborns with TPN having higher C0 compared to term, female, Black, and infants without TPN (Figures 2E–H). For leucine-Isoleucine, levels initially decreased except for newborns with TPN, and then increased after 30 h. Compared to term infants, preterm infants had higher leucine-isoleucine level in the first 30 h (*P* < 0.001) and lower levels after 48 h (*P* = 0.01). Newborns with TPN had higher leucine-isoleucine than newborns without TPN in the first 120 h (Figures 2I–L). For C18:1, preterm and term newborns had similarly increasing levels until 72 h, after which levels continued to increase and then plateaued for term newborns, while levels for preterm newborns plateaued and then slightly decreased after 96 h. Females and infants with TPN had lower C18:1 levels compared to males and infants without TPN (Figures 2M–P).

### Correlation of AaBC-Related Differences to False-Positive Results

We identified AaBC-related differences for 56% (23 of 41, Cohen's *d* > 0.2) of the metabolites when comparing levels between the early or late collection-group to the standard group (Figure 3). Seven of the 23 metabolites are primary NBS markers for detection of 11 metabolic disorders for which we had information on false-positive cases (Table 1). We reasoned that a disease marker elevated in the early-collection group could also lead to a higher number of false-positives in this group. In turn, a marker with significantly lower physiological levels at early AaBC could lead to a relatively lower number of false-positives compared to later collection-time groups. We defined this correlation as consistent with this hypothesis, or as inconsistent if this correlation was not found (Supplementary Table 3). Compared to the standard group, the early-collection group had elevated levels of PKU marker phenylalanine (Cohen's *d* = 0.55), IVA marker C5 (Cohen's *d* = 0.24), MMA marker C3 (Cohen's *d* = 0.23), and CTD marker C0 (Cohen's *d* = 0.23). These findings correlated with higher false-positive rates for PKU (*P* < 0.05), IVA (*P* < 0.05), and MMA (*P* < 0.001), and lower false-positive rate for CTD (*P* < 0.001, decreased C0 level in CTD patients) in the early-collection group. The late-collection group had decreased levels of phenylalanine (Cohen's d = −0.41) and citrulline (Cohen's *d* = −0.28), which was associated with fewer false-positive cases for PKU (*P* < 0.05) and more false-positives for OTCD (*P* < 0.001, decreased citrulline level in OTCD patients). There were also inconsistent results including an unexpectedly lower false-positive rate for IVA, and a higher false-positive rate for CITR and VLCADD in the late-collection groups despite the elevated C5, decreased citrulline and decreased C14:1 levels in this group.

## Discussion

Here we used population-level mass spectrometry screening data to systematically examine postnatal metabolic changes and whether AaBC could impact the performance of newborn screening for selected metabolic diseases on the RUSP (18). We followed a stringent study design by controlling for the influence from the important covariates of birth weight and gestational age in the analysis of metabolite levels across different AaBC timepoints. A cluster analysis of 41 metabolites reported for 410,918 screen-negative infants in relation to their AaBC revealed two large metabolite groups characterized by either decreasing or increasing levels after birth (Figure 1). While largely exploratory, this analysis could shed new light on early postnatal metabolism and the dynamic changes of individual screening markers. For example, phenylalanine levels markedly decreased within 48 h after birth, which may require different cutoff values in PKU screening based on the infant's AaBC. For the C3/C2 ratio, a screening marker for MMA and PA, levels decreased after 120 h (Supplementary Figure 2). This finding could explain the discrepancy in testing of two babies affected with PA and MMA, respectively. In each case, the initial NBS test showed a positive result while a second confirmatory test several days later was found negative. At the time it was not known that the first test was a true-positive while the second was a false-negative (32).

We next studied the influence on metabolite levels for several clinical variables (GA, sex, ethnicity and TPN status) and their relationship with AaBC (Figure 2). At AaBC under 72 h, the four selected metabolites displayed similar patterns in relation to differences in GA, sex and ethnicity, while patterns changed at AaBC after 96 h. A potential cause for these changes could be limitations in sample size, which decreased with increasing AaBC (97–120 h: *n* = 1657; 121–144 h: *n* = 627; 145–168 h: *n* = 326) leading to increased variance of the estimated mean. Other reasons for the metabolic pattern changes related to later AaBC may be the postnatal advance and increasing environmental changes, or differences related to race/ethnicity status (17, 25). We found that White infants had a tendency for later blood collection (26.0% between 24–48 h, 32.6% between 49–168 h, *P* < 0.001), which could lead to differences in metabolic patterns in later AaBC groups. A larger sample size is required to explore these questions and to control for the influence of the different variables. In contrast to the other covariates studied, TPN was associated with different metabolic patterns in relation to AaBC, and particularly for blood sampling before 24 h (Figures 2D,H,L,P). While these differences could be caused directly by TPN, there may also be other confounding factors related to TPN such as preterm birth or an unknown disease status (33). We only included term infants (37–41 weeks) with a normal birth weight (2,500 g to 4,000 g) in the TPN analysis based on our finding of a smaller fraction of newborns with TPN amongst term infants (0.45%) compared to preterm infants (12.13%).

We reasoned that the AaBC-related differences identified for 56% of the metabolites (Figure 3) could lead to false-positive screens. We selected 11 diseases with frequent false-positive screening results (Table 1). Analysis of false-positives for some of these diseases indicated AaBC-related differences, which correlated with differences in marker levels discovered in the respective collection groups. For example, infants in the early-collection group (12–23 h) were more likely false-positive in PKU, MMA and IVA screening, which correlated with the elevated phenylalanine, C3 and C5 levels in screen-negatives in this group. Similarly, higher levels of C0 in the early-collection group correlated with fewer false-positives for CTD (decreased C0 marker) in this group. C0 levels were found to be lower in the standard group (24–48 h) potentially contributing to a relatively higher false-positive rate for CTD in this group (Supplementary Figure 3). In the late-collection group (49–168 h), infants were more likely false-positives for OTCD, which correlated with the lower citrulline levels in screen-negatives in this group. In contrast, we did not find significantly more false positives for HCY and PA in the early-collection groups which was likely due to the smaller sample size of false-positives for these two disorders. Another unexplained result was the high false-positive rate for CITR and VLCADD, and the low false-positive rate for IVA in the late-collection group despite the decreased citrulline and C14:1 levels, and increased C5 levels amongst screen-negatives in this group. It is possible that marker levels may have been adjusted at late AaBC for these diseases; infants could have received blood transfusions, particularly in the late-collection group; or the infant's condition, which contributed to false-positive results, had precluded collection of the first newborn screening specimen before 48 h of life.

Here we identified an association between MS/MS disease markers and timing of blood collection, and showed that these differences could lead to false-positive screens for some disorders (Table 1). Previous studies suggested different cutoff values according to AaBC for hypothyroidism screening (7), or polynomial regression models to adjust metabolite levels and ratios for age at collection and birth weight in order to reduce false-positive results for lysosomal disorders (16). As shown in Figure 2, AaBC did not have a linear relationship with metabolite levels and different patterns were found for different metabolites. The association between metabolite levels and AaBC was also dependent on other confounding variables, of which GA and TPN had the largest influence. For example, both AaBC and GA are associated with differences in tyrosine levels. While tyrosine levels were higher at standard AaBC (24–48 h) compared to late AaBC (49–72 h) for both preterm (*P* < 0.001) and for term infants (*P* < 0.001), preterm infants had significantly higher tyrosine levels than term infants at both AaBC timepoints (Supplementary Figure 4). Relationships between AaBC and other covariates can be explored for all metabolites using an online tool accompanying this study (http://rusptools.shinyapps.io/AaBC/). Our results indicate that relying on cutoff values or regression model adjustment for metabolite levels based on AaBC could have limitations. Development of novel data mining models that incorporate all screening metabolites and clinical variables could further our understanding of complex metabolite-covariate relationships and improve prediction of metabolic disease status (34). Implementing these new tools and approaches is challenging and relies on collaborative efforts between NBS programs worldwide (35).

## Data Availability Statement

The data analyzed in this study is subject to the following licenses/restrictions: The data used in this study were obtained from the California Biobank Program (CBP) under SIS request 886. The data can be obtained by others after submitting a new request to the CBP coordinator. Requests to access these datasets should be directed to CaliforniaBiobank@cdph.ca.gov.

## Ethics Statement

The studies involving human participants were reviewed and approved by the institutional review boards at Yale University, Stanford University, and the State of California Committee for the Protection of Human Subjects. Written informed consent from the participants' legal guardian/next of kin was not required to participate in this study in accordance with the national legislation and the institutional requirements.

## Author Contributions

GP and CS designed the study and wrote the manuscript. GP and YT performed the statistical analysis. TC, HZ, and CS provided input on data analysis and interpretation. All authors edited and approved the manuscript.

## Conflict of Interest

The authors declare that the research was conducted in the absence of any commercial or financial relationships that could be construed as a potential conflict of interest.
