# Supplementary material for: Timing of Newborn Blood Collection Alters Metabolic Disease Screening Performance
Source: Front Pediatr. 2021 Jan 20;8:623184. doi: 10.3389/fped.2020.623184 (PMC7854909; doi:10.3389/fped.2020.623184)

Supplementary Material

# Supplementary Figures and Tables

## Supplementary Figures


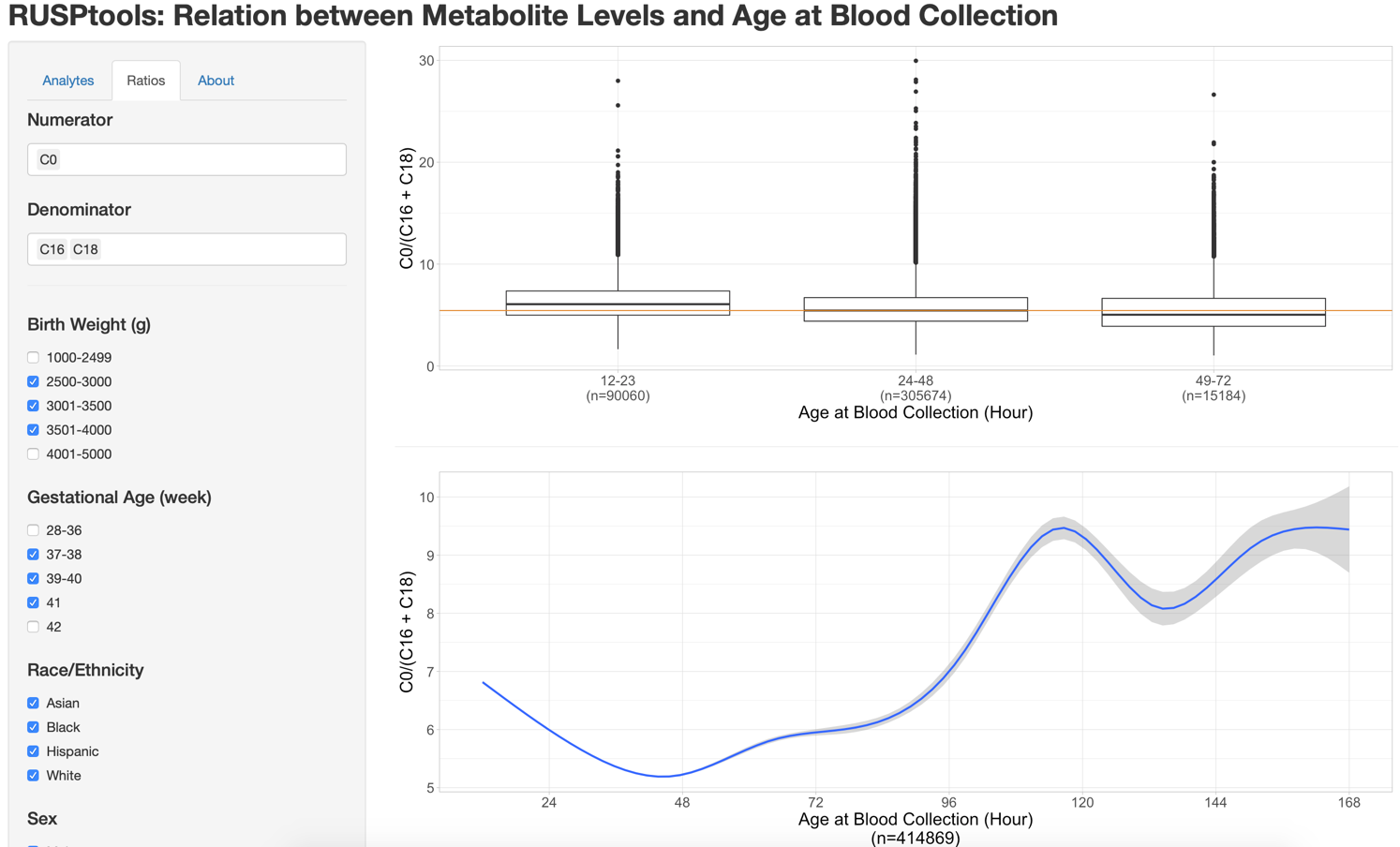


**Supplementary Figure 1.** **Screenshot of the online tool.** The figures show the results of C0/(C16+C18). The boxplot on the top right shows the distribution of C0/(C16+C18) in three categories: AaBC between 12-23 hours, AaBC between 24-48 hours, and AaBC between 49-72 hours. The line figure on the bottom right shows the mean of C0/(C16+C18) estimated from generalized additive model and the shading is the 95% confidence interval of the mean estimation from 12 to 168 hours. Users can use the left panel to change the analytes and scenarios.

**Supplementary Figure 2.** **Relationship between C3/C2 levels and age at blood collection.** Only term newborns (37-41 weeks) with birth weight between 2500 and 4000g and without TPN (n= 414,869) were included in the analysis. The solid smoothed line is the mean estimated from generalized additive model and the shading shows the 95% confidence interval of the mean estimation.

**Supplementary Figure 3. Relationship between C0 levels and age at blood collection.** Only term newborns (37-41 weeks) with birth weight between 2500 and 4000g and without TPN (n= 414,869) were included in the analysis. The solid smoothed line is the mean estimated from generalized additive model and the shading shows the 95% confidence interval of the mean estimation.

**Supplementary Figure 4.** **Boxplot of tyrosine levels for preterm and term newborns within different age at blood collection groups.** The red and blue dashed lines show the median of tyrosine levels for preterm and term infants, respectively, with recommended age at blood collection. There are 16,922 preterm infants with AaBC between 24 to 48 hours and 2,242 preterm infants with AaBC between 49 to 72 hours. There are 340,173 term infants with AaBC between 24 to 48 hours and 17,599 term infants with AaBC between 49 to 72 hours. Only infants without TPN were included in the analysis.

## Supplementary Tables

**Supplementary Table 1. Participant and sub-group demographics.**

| Variables | Sample Size | Percentage |
| --- | --- | --- |
| GA (week) | |  |
| <=27* | 457 | 0.09% |
| 28-36 | 28,908 | 5.74% |
| 37-38 | 121,048 | 24.02% |
| 39-40 | 313,548 | 62.22% |
| 41 | 37,772 | 7.50% |
| 42 | 2,069 | 0.41% |
| >=43* | 133 | 0.03% |
| BW (g) | |  |
| <1000* | 605 | 0.12% |
| 1000-2499 | 20,972 | 4.16% |
| 2500-3000 | 90,050 | 17.87% |
| 3001-3500 | 208,065 | 41.29% |
| 3501-4000 | 142,456 | 28.27% |
| 4001-5000 | 41,120 | 8.16% |
| >5000* | 667 | 0.13% |
| Sex | |  |
| Male | 261,343 | 51.86% |
| Female | 241,113 | 47.85% |
| NA | 1,479 | 0.29% |
| Race/Ethnicity | |  |
| Asian | 73,825 | 14.65% |
| Black | 33,832 | 6.71% |
| Hispanic | 251,430 | 49.89% |
| White | 134,103 | 26.61% |
| OtherUnknown | 10,745 | 2.13% |
| TPN | |  |
| No | 492,097 | 97.65% |
| Yes | 5,943 | 1.18% |
| Unknown | 5,895 | 1.17% |
| AaBC(Hour) | |  |
| <12* | 272 | 0.05% |
| 12-23 | 106,314 | 21.10% |
| 24-48 | 366,598 | 72.75% |
| 49-168 | 28,987 | 5.75% |
| >168* | 1,317 | 0.26% |
| Unknown* | 447 | 0.09% |
| *The newborns in these categories were removed from the analysis. | |  |

**Supplementary Table 2.** Sample size and percentage of samples in three different age at blood collection categories for different gestational age and TPN groups.

|  |  | Age at Blood Collection (Hour) | | |
| --- | --- | --- | --- | --- |
|  |  | 12-23 | 24-48 | 49-168 |
| Gestational Age* | Preterm | 3,790 (15.50%) | 16,922 (69.22%) | 3,735 (15.28%) |
|  | Term | 100,246 (21.66%) | 340,173 (73.50%) | 22,387 (4.84%) |
|  | Post term | 404 (20.15%) | 1,442 (71.92%) | 159 (7.93%) |
| TPN** | No | 90,060 (21.71%) | 305,674 (73.68%) | 19,135 (4.61%) |
|  | Yes | 112 (6.64%) | 1,039 (61.63%) | 535 (31.73%) |

* We removed newborns with position and unknown TPN status without controlling birth weight during gestational age analysis, which included 489,258 newborns.

** We only included newborns with known TPN status, GA between 37-41 weeks and BW between 2500-4000g to control the influence from GA and BW in TPN analysis, which included 416,555 newborns.

**Supplementary Table 3.** Definition of consistent and inconsistent in false positive comparison.

|  |  | **Consistent** | | **Inconsistent** | |
| --- | --- | --- | --- | --- | --- |
|  |  | Cohen's d | | Cohen's d | |
|  |  | <-0.2 | >0.2 | <-0.2 | >0.2 |
| Metabolite Changes in Patients | Increased | Less FP | More FP | More FP | Less FP |
|  | Decreased | More FP | Less FP | Less FP | More FP |

# User guide for interpretive analysis of metabolite levels and age at blood collection

This web-based tool was developed to enables studies of the association between metabolite levels and age and blood collection and other covariates (<https://rusptools.shinyapps.io/AaBC/>). We propose this tool could be of primary interest to NBS reference laboratories for evaluating data from MS/MS screen-positive cases.

**Instructions**

Select **Analytes** or **Ratios** from the left sidebar of the window.

**Analytes** enables the selection and analysis of individual analytes in relation to different covariates parameters. C3 was chosen by default.

**Ratios** enables the selection and analysis of metabolite ratios between all 41 metabolites. A specific numerator and denominator can be chosen to calculate a metabolite ratio. By default, C3 is the numerator and C2 is the denominator. If more than one metabolite is selected for either the numerator or the denominator, the summation of selected metabolite levels will be used. For example, if C0 is selected for numerator and both C16 and C18 are selected for denominator, the ratio of C0/(C16+C18) will be investigated.

**Birth Weight**, **Gestational Age**, **Race/Ethnicity**, **Sex**, and **TPN** are covariates that can be selected to be included in the metabolite analysis. For each covariate, users can use the checkbox to include or exclude groups of newborns based on specific values for each covariate. For example, the tool only includes newborns with birth weight between 2500g and 4000g by default (**Birth Weight** checkbox 2500-3000, 3001-3500, and 3501-4000). To analyze infants in the birth weight range of 3501g and 5000g, users would deselect checkbox 2500-3000 and 3001-3500 and select 4001-5000.

Click **Submit** on the bottom of left side bar after selecting either **Analytes** or **Ratios** and specific covariates. NOTE: The online tool will not make any changes to the figures shown on the right during step 1 and 2 until **Submit** is clicked.

**Select comparing groups** provides a function to compare **Analyte** or **Ratio** differences between infants with different covariates. By default, **No Comparison** is selected under **Select comparing groups**. User can select Sex, Birth Weight, Gestational Age, Race, or TPN to compare **Analyte** or **Ratio** differences between infants with the selected covariates. For example, selecting Birth Weight under **Select comparing groups**, the tool will compare the **Analyte** or **Ratio** differences between infants with different birth weights as shown below. The boxplot on the top of right panel shows the level difference of C3 between 3 birth weight groups in three AaBC categories: 12-23, 24-48, and 48-72. The smooth line on the bottom of the left panel shows the changes of C3 levels across AaBC in the 3 birth weight groups.


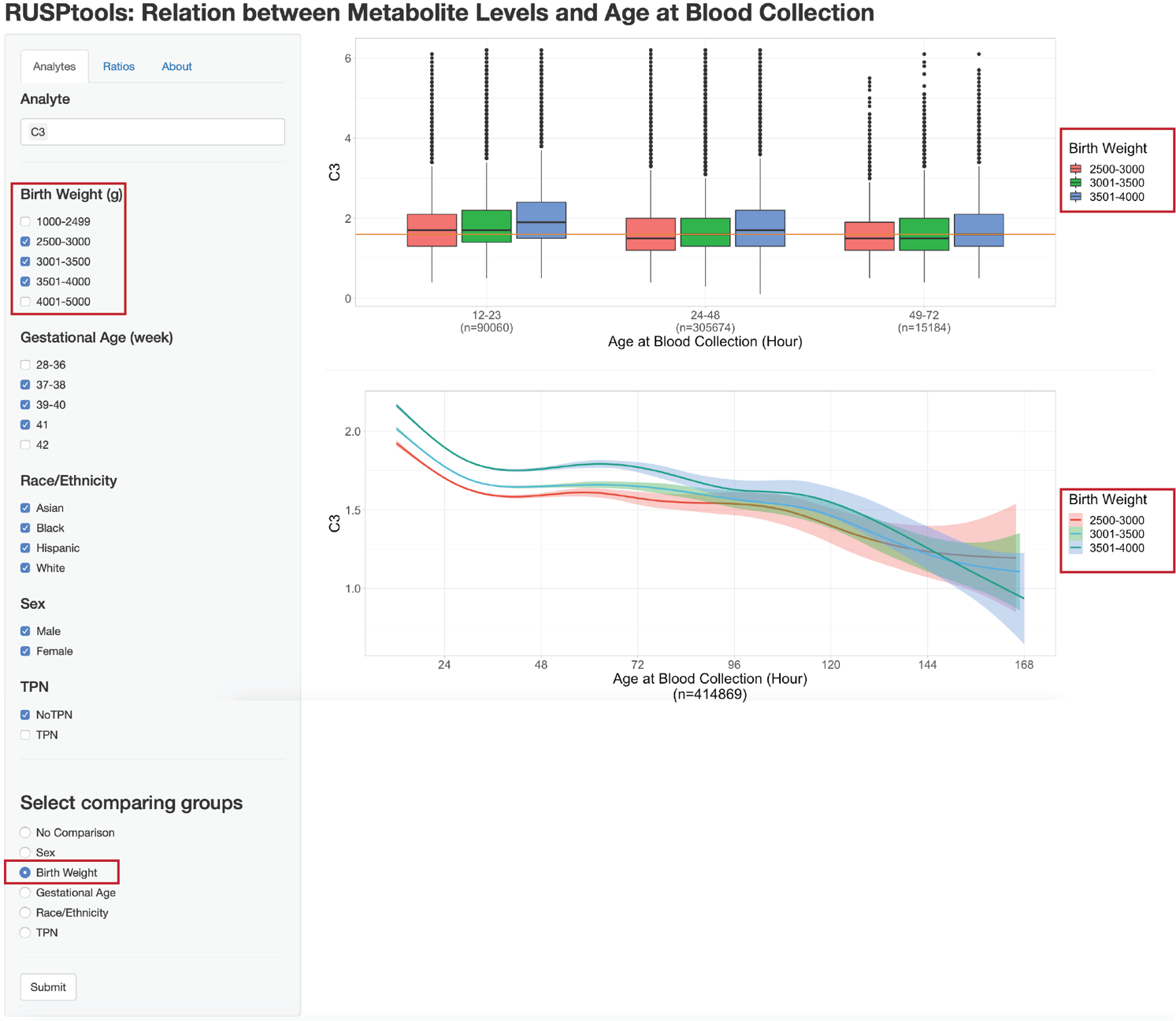

Supplement: Supplementary file 1 [file Data_Sheet_1.docx]
